# The use of indocyanine green and near-infrared fluorescence in the detection of metastatic lymph nodes during oesophageal and gastric cancer resection: a systematic review and meta-analysis

**DOI:** 10.1007/s00464-025-11703-7

**Published:** 2025-04-18

**Authors:** Naim Slim, Deepika Anbu, Ara Darzi, Daniel S. Elson, Christopher J. Peters

**Affiliations:** 1 Department of Surgery & Cancer, Imperial College London, London, UK; 2 Imperial College Healthcare NHS Trust, London, UK; 3 Hamlyn Centre, Imperial College London, London, UK; 4 Academic Surgical Unit, Institute of Global Health Innovation, Department of Surgery & Cancer, St. Mary’s Hospital, Imperial College London, 10 th Floor, Queen Elizabeth the Queen Mother Building, Praed Street, London, W2 1 NY UK

**Keywords:** ICG, Near-infrared fluorescence, Image-guided surgery, Lymph nodes, Gastric cancer, Oesophageal cancer

## Abstract

**Background:**

Lymph node status is one of the most important prognosticating factors for patients afflicted by oesophageal cancer (OC) and gastric cancer (GC), and lymphadenectomy during surgery is therefore an essential step to ensure complete oncological resection and accurate disease staging. Intraoperative lymph node visualisation using near-infrared fluorescence (NIRF) and indocyanine green (ICG) tracing has been postulated to improve the overall lymph node yield, and to ensure the appropriate radicality, but its usefulness in the detection of metastatic lymph nodes remains unclear.

**Methods:**

We conducted a systematic review and meta-analysis of the relevant literature to ascertain the accuracy of ICG-guided lymphadenectomy in the detection of metastatic nodes in OC and GC. The primary outcomes were the sensitivity, specificity and diagnostic odds ratio of ICG-guided lymphadenectomy. Secondary outcomes included measurement of the effect of prior neoadjuvant chemotherapy (NAC), tumour characteristics and method of ICG administration. Summary receiver operator characteristic (SROC) curves were built to illustrate the relationship between the sensitivity of ICG and false positive rate.

**Results:**

From an initial search of 6,302 articles, 15 studies met the criteria for inclusion, incorporating 4,004 patients. The pooled sensitivity for metastatic node detection was 69.1% (95% CI 56.5–79.3%), specificity 47.4% (38.0–56.9%), and DOR 2.02 (1.40–2.92). The SROC curve for diagnostic test accuracy yielded an area under the curve of 0.60. The use of NAC adversely affected the sensitivity of ICG 74.7% [59.2–85.8%] without NAC; 52.8% [43.6–61.9%] with NAC, *p* = 0.018). No significant difference in efficacy was demonstrated between pathological ‘T’ stage, or ICG administration method.

**Conclusion:**

Our findings suggest that the oncological benefits of NIRF and ICG in the context of lymphadenectomy in OC and GC are limited, and that surgeons risk omitting a significant proportion of metastatic nodes if this technique is solely relied upon.

**Supplementary Information:**

The online version contains supplementary material available at 10.1007/s00464-025-11703-7.

Despite advances in the diagnosis and treatment of oesophageal cancer (OC) and gastric cancer (GC), the long-term prognosis for patients afflicted by these cancers remains poor, with a five-year survival rate of 15–20% [1]. The quiescence of symptoms in the earlier phases of these cancers accounts for the fact that over half of patients have metastatic disease at the time of presentation [2]. Lymph node status is one of the most important factors for disease prognostication; the five-year survival rate for patients with no nodal disease is approximately 50–60%, and this falls dramatically to 6–9% amongst patients with two or more involved lymph nodes [3]. Lymphadenectomy is, therefore, an essential step to ensure complete oncological resection and accuracy of disease staging.

In spite of this, there is a distinct lack of consensus regarding the optimal strategy of lymphadenectomy during oesophagectomy and gastrectomy [4]. The pattern of lymph node distribution, and indeed metastases, is highly variable; it has been postulated that tumour location, invasion depth (‘T’ stage), history of neoadjuvant treatment and underlying tumour biology all influence the presence of, and pattern of, lymph node metastases [5, 6]. There is an equipoise between balancing oncological completeness through radicality of surgery, whilst attenuating the risk of complications—as such, there is considerable variation in practice when it comes to performing lymphadenectomy [7].

In the pursuit of enhancing the effectiveness of lymphadenectomy, image-guided techniques using near-infrared fluorescence (NIRF) with indocyanine green (ICG) have been developed. Such techniques have been demonstrated to improve the overall yield of lymph nodes obtained during lymphadenectomy [8, 9] but the usefulness of ICG in the detection of metastatic lymph nodes remains unclear.

This paper aims to systematically review the efficacy of ICG in the context of image-guided lymphadenectomy in oesophageal and gastric cancer resection, with the specific aim of establishing its usefulness in the detection of lymph node metastases.

## Materials and methods

### Overview

We performed a systematic review and meta-analysis of studies on the utility of ICG in the detection of lymph node metastases in the context of oesophagectomy and gastrectomy performed with curative intent. This review was reported according to The Preferred Reporting Items for Systematic Review and Meta-Analysis (PRISMA) guidelines [10], and was registered with PROSPERO (ID: CRD42024515908).

### Search strategy, selection criteria and data extraction

A systematic review was conducted to ascertain the sensitivity, specificity, and diagnostic odds ratio of ICG-guided lymphadenectomy in the detection of lymph node metastases. A literature search was conducted on the EMBASE, MEDLINE and Cochrane databases from 1 st January 1950 to November 2024 with the search terms as follows: (“cancer” AND “lymph node”) AND (“optic*” OR “spectroscop*” OR “*fluorescence/” OR “diffuse reflectance” OR “Raman” OR “scattering” OR “laser” OR “infrared” OR “ICG” OR “Indocyanine Green/”). Full-text review was subsequently performed to filter for studies incorporating oesophageal or gastric cancer resection, with near-infrared fluorescence and ICG tracing with near-infrared fluorescence. The inclusion criteria for article selection were as follows: (a) prospective or retrospective study of elective resectional oesophageal or gastric surgery cases, (b) with or without neoadjuvant chemoradiotherapy or previous endoscopic treatment, and (c) involving the use of NIRF-guided lymphadenectomy using ICG.

Included studies were required to demonstrate classification of harvested nodes as ‘fluorescent’ or ‘non-fluorescent’ at the time of surgery using a dedicated NIRF video device, and subsequent confirmation of pathological status of each node (fluorescent or not) by a dedicated pathologist, thereby providing summary statistics for diagnostic performance. Only studies that reported radical lymphadenectomy in keeping with their standard oncological practice were included; that is to say, papers that described metastatic lymph node yield within the context of limited sentinel node biopsy only, or for organ preserving surgery, were excluded from the analysis.

We excluded case series of fewer than ten patients, as well as studies that included emergency cases, palliative surgery cases, or redo (salvage) surgery cases where such patients could not be excluded from the analysis. Cases whereby lymphadenectomy was performed with a tracer other than ICG, or with a non-standard formulation (such as that bound to human soluble albumin), or with an optical method other than NIRF were also excluded.

A dedicated online database was developed for data extraction. Data pertaining to the following were extracted: (a) study characteristics: study aims; study design; study duration, (b) patient demographics: number of patients, broken down by sex; age; body mass index (BMI); American Society of Anaesthesiologists (ASA) grade, (c) cancer details: location (oesophageal/junctional or gastric); anthropomorphic features (length, circumference); previous neoadjuvant chemotherapy or endoscopic therapy; clinical staging, (d) operative details: operative approach; extent of lymphadenectomy; access (open, laparoscopic or robotic); operative time; complication data, (e) post-operative pathology: histology; pathological stage, (f) lymph node visualisation: ICG administration details (method, technique, dose, concentration, timing of administration); verification of ICG fluorescence, and (g) outcomes: total lymph node yield, total metastatic lymph node yield, ICG positive metastatic nodes (true positive—TP), ICG positive non-metastatic lymph nodes (false positive—FP), ICG negative metastatic nodes (false negative—FN) and ICG negative non-metastatic nodes (true negative—TN). Furthermore, the risk of bias in each study was assessed using the Diagnostic Precision Study Quality Assessment Tool (QUADAS- 2). Data was independently extracted by two reviewers (NS and DA), with resolution of disagreements by discussion with the senior author (CP).

The primary outcomes of this study were the pooled sensitivity (probability that a node would be ICG positive if it is metastatic), specificity (probability that a node would be ICG negative if it is not-metastatic) and diagnostic odds ratio (DOR—the odds of a fluorescent node if metastatic, relative to the odds of a fluorescent node if non-metastatic). These were calculated based on the studies’ TP, TN, FP, and FN rates. Sensitivity was calculated as TP/(TP + FN). Specificity was calculated as TN/(TN + FP). DOR was calculated as (TP × TN)/(FP × FN). Meta-analysis was performed with logit transformed data. Meta-regression was performed to examine the effect of the average lymph node yield reported in each study. Secondary outcome measures included measuring the effect of neoadjuvant chemotherapy (NAC); tumour characteristics, such as the location of the tumour—oesophageal or gastric—and the tumour invasion depth (or ‘T’ stage); and practical considerations, such as ICG dose and timing of administration, on the pooled diagnostic outcomes.

Summary receiver operator characteristic (SROC) curves were built to illustrate the relationship between the sensitivity of ICG and the false positive rate (the probability that a node is fluorescent yet non-metastatic, defined as 1-specificity). The diagnostic performance of ICG was deemed more favourable as the area under the curve (AUC) approached 1. Studies were assessed for heterogeneity by *χ*^2^ testing, with *I*^2^ greater than 75% deemed as high heterogeneity. Statistical analysis was performed with the ‘meta’ packages in *R* [Version 4.3.2, R Foundation for Statistical Computing, Vienna, Austria] [11].

## Results

**Fig. 1:**
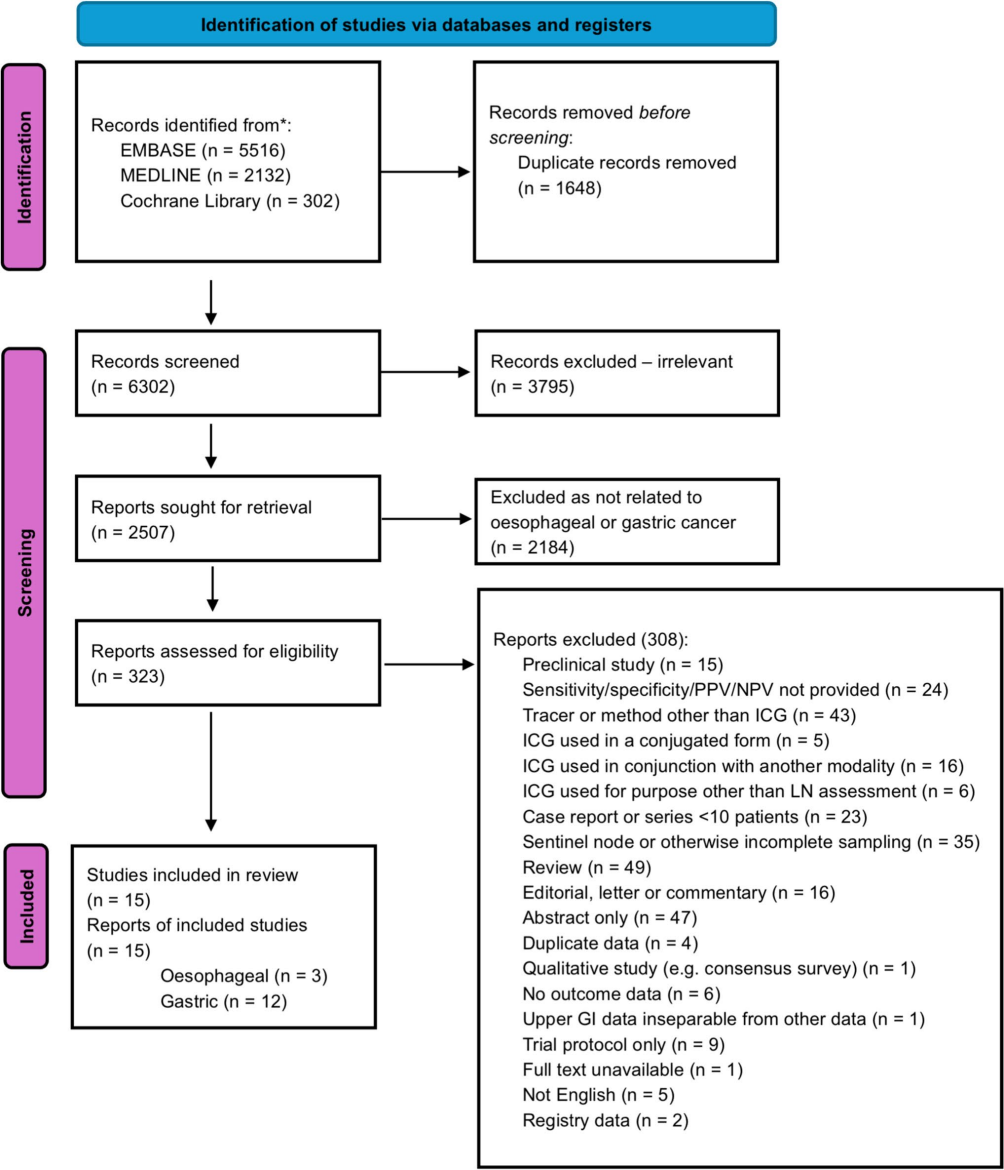
PRISMA diagram showing selection of records included in review

A total of 6302 articles were identified at initial search after removal of duplicates. 324 papers were relevant for full text review, and 15 papers ultimately met the criteria for inclusion in the review, as detailed in Fig. 1. Twelve studies [8, 12–22] described the use of ICG during gastrectomy, and three studies [23–25] described the use of ICG during oesophagectomy. A total of 4004 patients were included in the analysis, of which 3850 had GC, and 154 had OC. The weighted average nodal harvest of 50.0 nodes per patient (range 25.7–72.8). The characteristics of these are detailed in Table 1. The QUADAS- 2 tool was used to assess the risk of bias, the results of which are provided in Table 2.

**Table 1:** List of included studies

Refs.	Authors	Year	Study design	Origin of study	Location of cancer	Number of patients	Age (mean* or median^)	Pre-op NAC	ICG admin method **	Average nodal yield per patient	Total LN	Metastatic LN	TP	FP	FN	TN
[8]	Chen et al	2020	RCT	China	Gastric	129	57.8*	NO	POSM	50.9	6513	728	410	3116	318	2669
[18]	Cianchi et al	2020	Cohort	Italy	Gastric	37	72.2*	YES	POSM	50.8	1881	150	79	640	71	1091
[21]	Iwata et al	2022	Prospective	Japan	Oesophageal	16	69^	YES	POSM	63.9	1019	19	12	204	7	796
[17]	Chen et al	2021	RCT	China	Gastric	259	58.9*	NO	Both	49.5	12,816	1025	617	6505	408	5286
[16]	Baiocchi et al	2020	Prospective	Italy	Gastric	13	69^	NO	Both	37.9	417	54	54	282	0	81
[22]	Shiomi et al	2023	Prospective	Japan	Oesophageal	54	69^	YES	POSM	55.3	2989	110	55	723	55	2156
[15]	Lee et al	2022	Retrospective	S. Korea	Gastric	74	56.1*	NO	POSM	72.8	5385	216	183	2888	33	2281
[14]	Park et al	2022	Prospective	S. Korea	Gastric	10***	69^	NO	IOSS	62.5	628	63	24	218	39	347
[23]	Wang et al	2022	Retrospective	China	Oesophageal	84	61.27*	YES	POSM	25.7	2164	124	53	847	71	1193
[13]	Jung et al	2021	Retrospective	S. Korea	Gastric	592	55^	NO	POSM	58.2	33,720	799	703	22,213	96	11,428
[12]	Roh et al	2020	Retrospective	S. Korea	Gastric	98	63.4*	NO	POSM	47.7	4671	9	8	3078	1	1584
[19]	Kim et al	2024	Retrospective	S. Korea	Gastric	2397	58.8*	NO	POSM	48.4	130,961	2808	2416	80,075	392	48,078
[20]	Tuan et al	2024	Prospective	Vietnam	Gastric	79	61.5*	NO	POSM	37.7	2992	194	176	2216	18	582
[21]	Tian et al	2024	RCT	China	Gastric	44	57.4*	NO	POSM	53.6	2360	229	98	1124	131	1007
[22]	Huang et al	2024	RCT	China	Gastric	118	63.0*	YES	IOSS	48.2	5687	383	230	2960	153	2344

**POSM Perioperative submucosal, *IOSS* Intraoperative subserosal

***One patient was excluded as they had a palliative resection

**Table 2:**
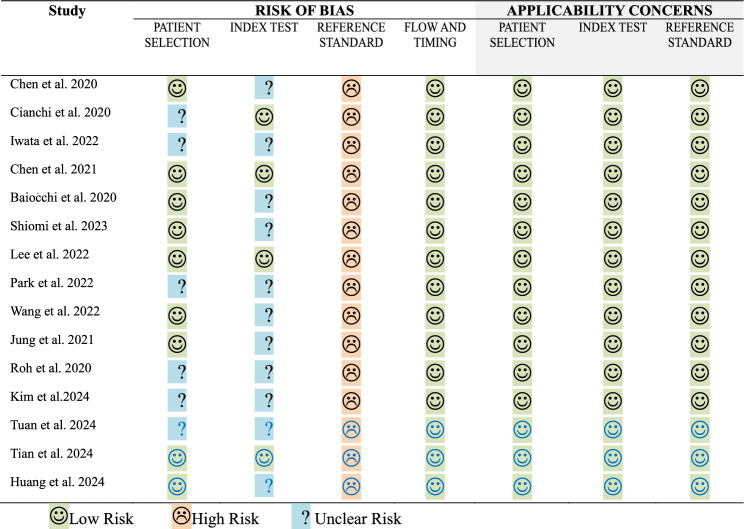
QUADAS- 2 Tool for assessment of risk of bias

### Overall outcomes

**Fig. 2:**
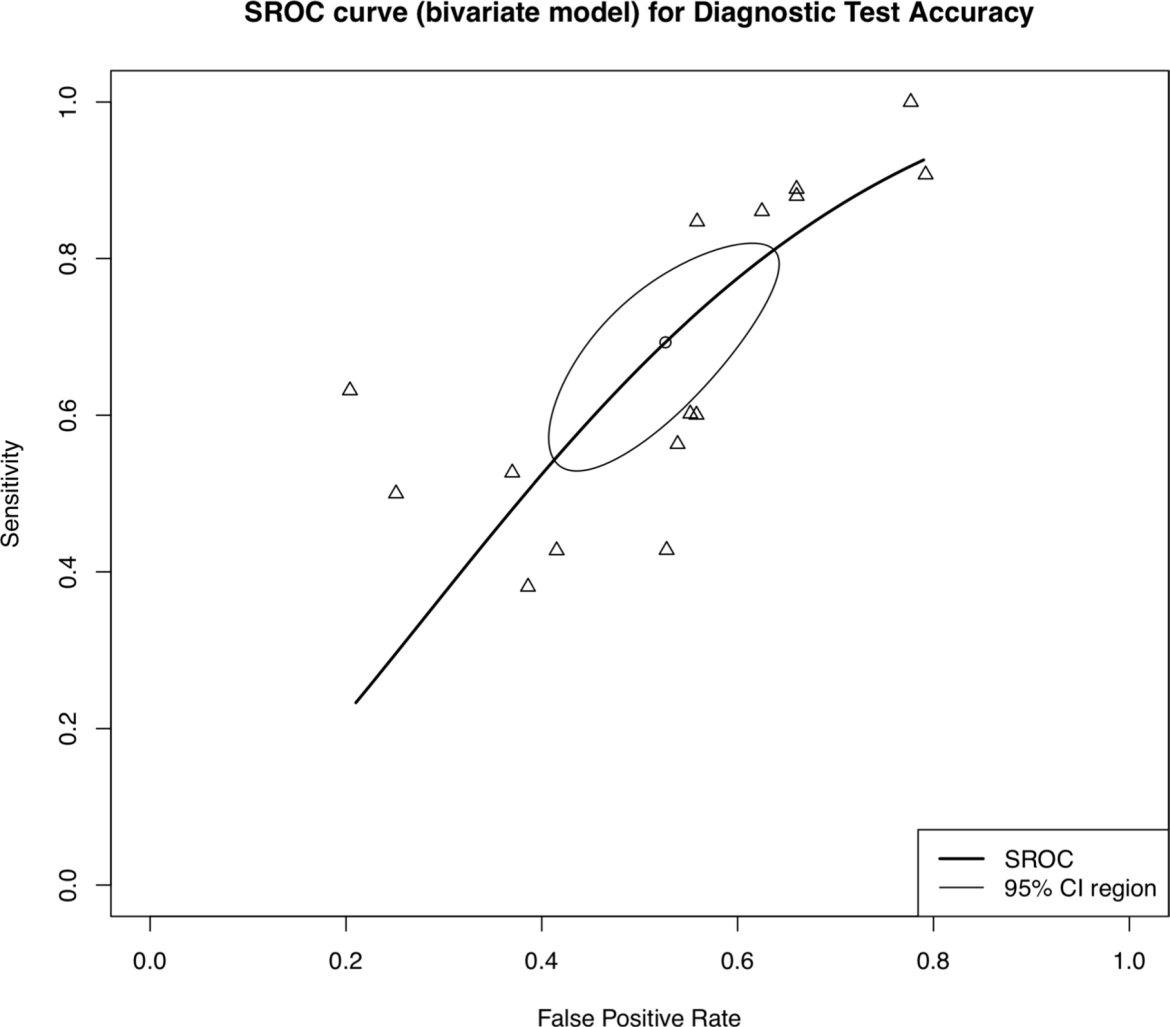
Summary receiver operator characteristic (SROC) curve demonstrating diagnostic test accuracy of ICG-guided lymphadenectomy

With regards to the detection of metastatic lymph nodes following administration of ICG, the pooled sensitivity for metastatic lymph node detection was 69.1% (95% CI 56.5–79.3%), the pooled specificity 47.4% (38.0–56.9%), and the pooled DOR was 2.02 (1.40–2.92). There was significant heterogeneity between studies, with an *I*^2^ value greater than 75% across all outcomes, further detailed in Table 3. Figure 2 illustrates the overall SROC curve for diagnostic test accuracy of ICG, with a calculated AUC of 0.60. The reported average lymph node yield did not affect the overall sensitivity, specificity or DOR following meta-regression analysis (*p* = 0.849; *p* = 0.141; *p* = 0.261, respectively).

**Table 3:** Meta-analysis outcomes by subgroups

Analysis	Outcome	Subgroup	Point estimate	95% CI	*P*-value	Heterogeneity		
						*I*^2^ (%)	Q	*p*-value
Overall	Sensitivity (%)		69.1	56.5–79.4	–	98.2	795.56	< 0.0001
	Specificity (%)		47.4	38.0–56.9	–	99.7	4018.53	< 0.0001
	DOR		2.02	1.40–2.92	–	96.5	403.55	< 0.0001
NAC	Sensitivity	NAC−	76.9	60.6–87.8		98.7	666.90	< 0.0001
	(%)	NAC +	52.8	13.1–68.1	**0.0094**	69.7	13.19	0.010
	Specificity	NAC−	38.6	31.1–46.7		99.3	1308.56	< 0.0001
	(%)	NAC +	64.9	51.6–76.3	**0.0010**	99.6	917.93	< 0.0001
	DOR	NAC−	2.06	1.25–3.39		97.5	358.83	< 0.0001
		NAC +	1.98	1.12–3.52	0.9234	87.5	32.03	< 0.0001
Location	Sensitivity	GC	73.4	59.1–84.0		98.5	727.09	0.000
	(%)	OC	48.0	40.3–55.8	**0.0028**	37.5	3.20	0.202
	Specificity	GC	41.0	33.6–48.9		99.4	1824.12	0.000
	(%)	OC	71.7	58.2–82.2	**0.0002**	99.0	200.77	0.000
	DOR	GC	1.91	1.28–2.87		97.1	381.39	< 0.0001
		OC	2.58	0.93–7.16	0.5934	91.0	22.15	< 0.0001
Tumour stage	Sensitivity	pT0—pT2	85.1	51.1–96.9		91.5	35.10	< 0.0001
	(%)	pT3—pT4	63.7	40.5–81.9	0.2345	97.9	144.21	< 0.0001
	Specificity	pT0—pT2	49.3	26.0–73.0		99.6	670.37	0.000
	(%)	pT3—pT4	54.6	36.7–71.3	0.7432	99.4	483.19	0.000
	DOR	pT0—pT2	5.24	1.39–19.7		88.5	26.12	< 0.0001
		pT3—pT4	1.92	0.95–3.91	0.1914	94.4	53.55	< 0.0001

*DOR* diagnostic odds ratio, *GC* gastric cancer, *NAC* neoadjuvant chemotherapy, *OC* oesophageal cancer

Bold *p*-values indicate statistical significance (*p*<0.05)

### Effect of neoadjuvant chemotherapy

**Fig. 3:**
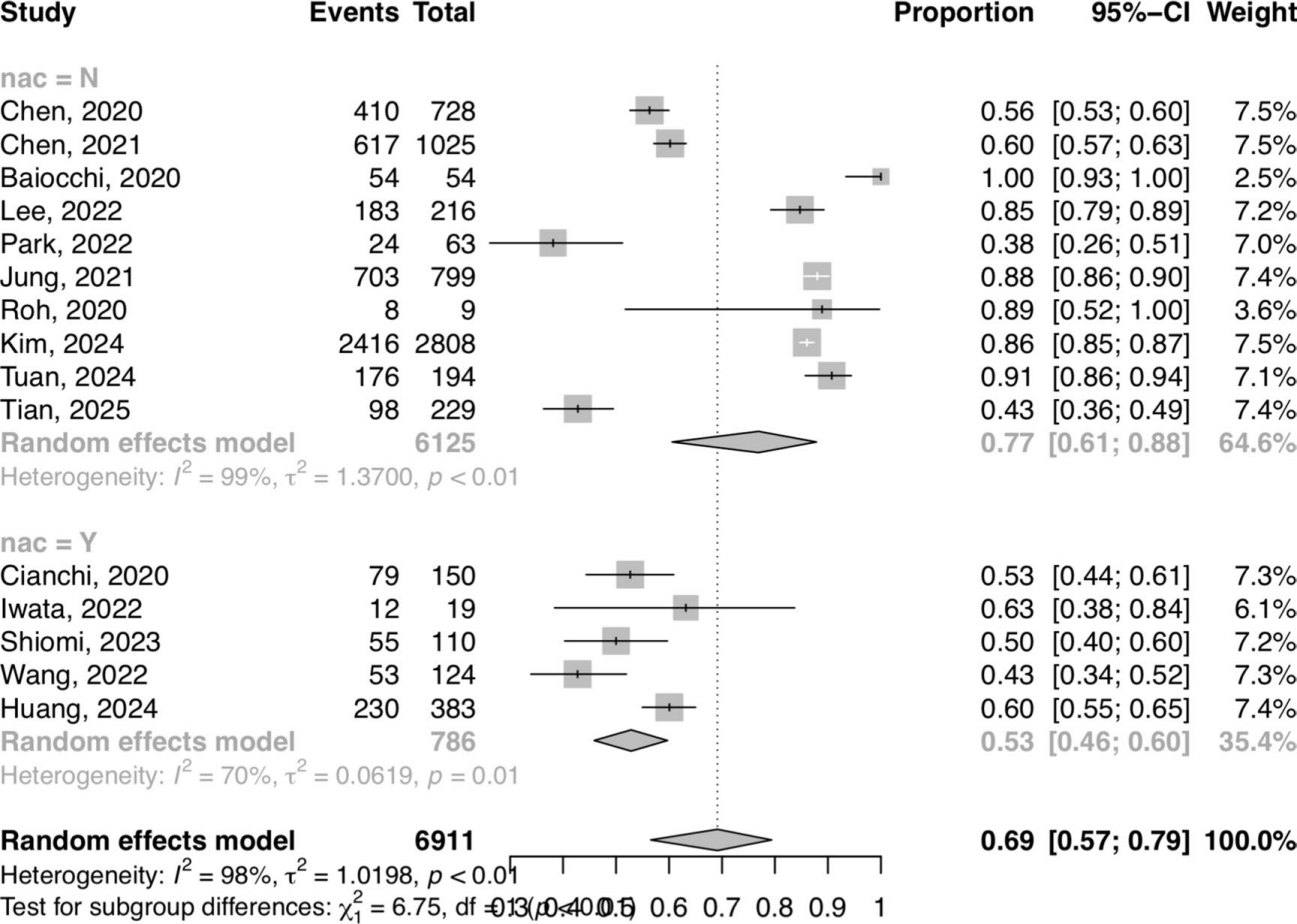
Forest plot detailing the effect of neoadjuvant chemotherapy (NAC) on sensitivity of metastatic lymph node detection with ICG

The sensitivity for metastatic lymph node detection in cohorts without prior NAC was 76.9% (95% CI 60.6–87.8%) and with prior NAC) 52.8% (43.6–61.9%), and this was statistically significant (*p* = 0.009). The specificity without prior NAC was 40.2% 38.6% (31.1–46.7%) and with prior NAC was 64.9% (51.6–76.3%), which was also statistically significant (*p* = 0.001). The DOR without NAC was 2.06 (1.25–3.39) and with NAC was 1.98 (1.12–3.52), and this did not differ significantly between groups (*p* = 0.923). Forest plots detailing the effect of NAC are shown in Fig. 3.

### Effect of tumour characteristics

Further sub-group analysis was undertaken to ascertain the effect of tumour location and stage. The sensitivity for metastatic nodal detection in GC was 73.4% (59.1–84.0%) and in OC 48.0% (40.3–55.8%), and this was statistically significant (*p* = 0.003). The specificity was 41.0% (33.6–48.9%) for GC and 71.1% (58.2–82.2%) for OC and this was also statistically significant (*p* < 0.001). The DOR did not differ significantly between groups (*p* = 0.593). The effect of the tumour pathological ‘T’ stage on metastatic lymph node detection was described in six studies (five [12–14, 17, 20] with GC patients and one [23] with OC patients), and no statistical significance was found between early (pT0—pT2) and advanced (pT3—pT4) cancers. The outcomes of these analyses are detailed in Table 3.

### Effect of variations in practical aspects of ICG administration

The two approaches described in the literature regarding the administration of ICG is the submucosal approach, whereby ICG is injected around the tumour endoscopically in the mucosal layer of the stomach or oesophagus prior to surgery, and the subserosal approach whereby ICG is injected beneath the serosa of the stomach during surgery. The overwhelming majority of studies reported preoperative submucosal injection as the preferred method of ICG administration in both OG and GC. A direct comparison between the submucosal and subserosal approaches in GC is made in one study—the FUGES- 019 trial [17], the results of which showed comparable sensitivity and specificity between the submucosal (62.2% sensitivity, 48.5% specificity) and subserosal (58.0% sensitivity, 41.2% specificity) approaches. One case series [14] described the use of intraoperative subserosal injection in ten GC patients, with a reported sensitivity, specificity, and DOR of 38.1%, 61.4%, and 0.98, respectively. Another case series [16] described the utilisation of ICG in their 13 GC patients with a 7:4 split between the submucosal and subserosal approach, and whilst they found no differences in overall nodal yield, the authors did not report the effect on the diagnostic value of ICG between approaches.

## Discussion

The pattern of lymph node spread in oesophageal and gastric cancer is complicated. It is related, at least in part, to the presence of a complex array of lymph channels that drain in a multidirectional manner [26, 27], and this provides some explanation regarding the variation of the location of lymph node metastases. Furthermore, the phenomenon of skip metastases, whereby nodal metastases are detected in anatomically distant nodes [28, 29], further complicates efforts to standardise the extent of optimal lymphadenectomy—a matter that remains controversial to this date. Currently, the consensus as per the American Joint Committee on Cancer (AJCC) is that a minimum of 15 lymph nodes during esophagectomy, and 16 for gastrectomy, are required for accurate nodal staging [30, 31], but the evidence base suggests that more extensive lymphadenectomy may improve overall survival [32, 33]. However, there is a need to balance the desire for oncological completeness through thorough lymphadenectomy, with the need to reduce the morbidity of radical surgery. This is of most importance when concerned with dissection of the lymphatics of the recurrent laryngeal nerve [34, 35] and subcarinal lymph nodes [36] during oesophagectomy, with the associated risk of nerve palsy and tracheal injury, respectively. At the time of writing, a large multi-centre observational study (the TIGER study—NCT03222895) is currently in progress, which will endeavour to elucidate patterns of lymph node metastases with a view to providing guidelines for improving lymph node staging in OC [37]. This may serve as a roadmap for a more tailored lymphadenectomy in the future.

Image-guided surgery using a tracer such as ICG has shown promise for tailoring lymphadenectomy, but there are several practical considerations that affect reliability in this setting. ICG is a diagnostic compound that first gained traction for clinical use in the context of measuring hepatic function in mammals in the 1960 s [38]. It fluoresces when stimulated by near-infrared light at a wavelength between 800 and 840 nm, and binds rapidly to plasma proteins upon intravenous injection [39], making it an ideal tracer for a variety of luminal structures that transmit the flow of plasma, such as blood vessels and lymphatic channels. As such, the wide variety of use cases has invariably led to the integration of fluorescent endoscopes in existing surgical endoscopic systems [40] which enable the visualisation of ICG by capturing the emitted fluorescence through specialised band-pass filters that are selective for the near-infrared wavelength range [41]. Clinically, image-guided surgery with ICG has been demonstrated in the assessment of perfusion [42, 43], the localisation of important anatomical structures [44, 45] and indeed the visualisation of lymphatics in several cancers, such as breast [46, 47], gynaecological [48–50], gastrointestinal [51–53], and urological [54, 55] cancers. However, for the purposes of lymph node visualisation, peritumoral injection, either by the submucosal or subserosal approach, is the preferred method of visualising the lymphatic chains as they drain from the tumour [56]. From a practical perspective, the efficacy of this technique is likely to be diminished in cases where the tumour is obstructing and impassable by an endoscope, or, paradoxically, where a tumour has exhibited an excellent response to NAC such that it cannot be located easily at endoscopy. The technique can also fail if the tracer is administered incorrectly; extraluminal spillage of ICG and subsequent obscuration of the view of the lymph node stations was reported in three of the included studies [8, 16, 17]. There is a learning curve associated with success of the technique; an earlier Japanese multicentre trial (JCOG0302) investigating the feasibility of sentinel node mapping using ICG was terminated early due to a high false-negative rate, with technical issues related to ICG administration and poor initial training at an institutional level deemed putative factors for technique failure [57, 58]. Furthermore, all the patients in the included studies that described endoscopic peritumoral injection were required to undergo endoscopy the day before the scheduled procedure; in the United Kingdom at least, admission to hospital is typically on the day of surgery [59] and therefore the routine adoption of this technique would add an additional step in the patient pathway, along with a resultant effect on hospital resources.

In this study, we found that the overall sensitivity of ICG-guided lymphadenectomy in the detection of metastatic lymph nodes in patients with oesophageal and gastric cancer was 69.1%, the overall specificity was 47.4%, and the overall DOR was 2.02. These results indicate that the usefulness of ICG-guided lymphadenectomy as a technique to identify metastatic lymph nodes is limited. There is the potential for missing a significant proportion of metastatic nodes if this technique is relied upon as a means of lymph node identification. ICG does not exhibit any preferential binding towards cancer cells [60]. The crux of the argument for the use of ICG with metastatic node detection rests with the theory that by mapping the nodes that drain directly from the tumour, it will lead to a greater detection of metastatic nodes through improvement in the overall nodal yield. This ultimately causes numerous nodes to fluoresce, with many false positives. Conversely, the flow of ICG can be impeded in instances whereby a node or its associated capillaries have been completely effaced by tumour [61], resulting in failure to fluoresce and a reduction in sensitivity.

Furthermore, the sensitivity fell to approximately 52.8% when sub-group analysis was performed to examine the effect of NAC. The majority of patients with OC and GC have, at least, locally advanced disease at the time of presentation [62, 63] and will therefore have received NAC as part of a multimodal treatment strategy. This therefore presents a dilemma for surgeons wishing to utilise ICG guidance during lymphadenectomy. The effect of NAC on the lymphovasculature, and ergo the rationale for the reduction in efficacy is poorly understood. In the SENTINA trial [64], it was observed that the accuracy of sentinel node biopsy in patients undergoing chemotherapy for node-positive breast cancer (using lymphoscintigraphy and radio-isotope colloid guidance) was reduced by approximately 20% after chemotherapy, and that chemotherapy-induced fibrosis of the lymphatic capillaries was postulated to play a role. More recently, Hara and colleagues [65] observed a significantly lower number of peritumoural lymphatic vessels in the lamina propria mucosa amongst patients receiving NAC following histological evaluation of 163 patients with squamous cell carcinoma of the oesophagus. As these observations were made on resected specimens, no comparison was drawn pre- and post-NAC, and as such these findings should be interpreted with caution, but nonetheless it could explain the impairment of peritumoral flow of ICG and warrants further research in this field.

We observed a difference in diagnostic performance between GC and OC, with the latter afflicted by poorer sensitivity (73.4% versus 48.0%, respectively). There may be technical factors related to the administration of ICG that could account for the difference—for example, challenges with peritumoral injection of ICG in the narrow oesophagus compared with the stomach. Furthermore, the ability to inject ICG in the serosal layer in the stomach cannot be replicated in the oesophagus owing to a lack of a true serosal layer [66]. We urge caution regarding the interpretation of this results, due to the limited number of studies in OC and the fact that all three of these observational studies involved patients who had NAC; a composite effect of NAC therefore could not be entirely ruled out. To date, only one RCT exists in OC conducted by Du et al. [67] comparing ICG versus placebo for mediastinal lymphadenectomy in a cohort of 40 patients with early SCC of the oesophagus of which none of whom had NAC, and reported a sensitivity of 100% and specificity of 65.9%. However, this was excluded from our meta-analysis because only the mediastinal lymph node stations were assessed in their trial protocol; the cervical and abdominal lymph node stations were omitted. Further high-quality studies are warranted to assess the true diagnostic performance of ICG in chemotherapy-naïve patients with OC.

We did not discern an appreciable effect of tumour ‘T’ stage on the efficacy of ICG-guided lymphadenectomy. However, due to the heterogeneity of the data, it was difficult to reliably stratify by individual pathological ‘T’ stages and it is possible that there may be an appreciable effect when individual stages are examined separately. In the study performed by Roh et al. [12], a high sensitivity (88.9%) was demonstrated in their cohort of 98 patients with early (pT1a-b) GC, but the overall burden of metastatic nodal disease was very low (nine metastatic nodes in total). Conversely, the research conducted by Jung et al. [13] found a dwindling effect in sensitivity with increasing pathological ‘T’ stage in GC, ranging from 100% for pT1a cancers, through to 64.5% to pT4 cancers. There is, therefore, the potential that the efficacy of ICG may be affected by the depth of tumour invasion, but further studies are warranted to elucidate its true effect.

Though we have demonstrated the potential shortcomings of ICG in this study, NIRF as an optical spectroscopic method remains a useful method for the visualisation of anatomical structures. Electromagnetic radiation in the NIR range exhibits deeper tissue penetration because it is less susceptible to absorption by (deoxy-)haemoglobin and water, as well as a favourable signal-to-noise ratio due to reduced tissue autofluorescence in this range [68]. These properties allow the enhancement of structures that would not otherwise be readily visible to the naked eye or by conventional white light endoscopy, such as lymph nodes embedded within adipose tissue. Where haptic feedback is reduced in the case of robotic or minimally invasive surgery, NIRF-guided lymphadenectomy can reduce the rate of lymph node ‘non-compliance’—the dissection of lymph node stations whereby no lymph nodes are found during histopathological analysis [8]. The sensitivity of this technique may be improved by utilising fluorophores that selectively bind to cancer cells; emerging evidence from preclinical studies suggest this may be achieved by conjugation of ICG with metal compound nanoparticles, such as ferritin [60] or copper sulphate [69], or by utilising dyes conjugated with antibodies that recognise cancer-associated receptors, such as EGFR and c-Met [70, 71]. These rely upon the extension of NIRF into the ‘second’ window (NIR-II), with a wavelength range of 1000–1700 nm—which, although confers more favourable tissue penetration and autofluorescence reduction than NIR-I, is yet to be integrated within commercial surgical systems due to the lack of NIR-II fluorophores approved for use in humans, as well as the cost of indium gallium arsenide (IgGaAs) cameras required for optimal sensitivity in the NIR-II range [72–74].

The results of this meta-analysis provide a less optimistic estimate of the performance of ICG-guided lymphadenectomy than prior studies. A recent meta-analysis performed by Jimenez-Lillo et al. [9] derived a pooled sensitivity of 89% and specificity of 15% in their meta-analysis of six studies detailing the use of ICG in OC. However, each study included a very small number of patients, often with single digit lymph node yields, and this affected the robustness of the data. Furthermore, the effects of tumour stage and NAC were not examined in this meta-analysis. Another recent meta-analysis examining the performance of ICG performed by Skubleny et al. [75] gave a similarly optimistic sensitivity of 87% and specificity of 100%, but once again did account for the effect of neoadjuvant chemotherapy, and was disproportionately weighted towards early GC, with T1 tumours comprising 80% of included cases. A meta-analysis performed by He et al. [76] examined the feasibility of sentinel node mapping in GC and derived a pooled sensitivity of 94% and specificity of 100% for detection of the sentinel nodes, but its applicability in the context of systemic lymphadenectomy is less established. More recent meta-analyses by Zhang et al. [77], Dong et al. [78], Deng et al. [79], Sposito et al. [80], and Yang et al. [81] focusing on the use of ICG in GC demonstrate an improved metastatic nodal yield and reduction in the likelihood of lymph node non-compliance, but do not comment on the performance of the technique with regard to metastatic nodal detection.

Our meta-analysis elucidates the diagnostic performance of ICG in OC and GC in a large cohort of patients, incorporating data from two randomised trials as part of the analysis. To the best of our knowledge, this is the first meta-analysis examining the effect of NAC on the diagnostic performance of ICG in the context of lymphadenectomy. We opted to examine the sensitivity, specificity and DOR using the individual nodal count, as we believe this level of granularity provides the best indication of diagnostic performance when compared to using the lymph node basin count; there were several occasions whereby individual metastatic lymph nodes were reported to be present outside of fluorescent basins, and this is likely to have a significant prognostic impact. Furthermore, the years of publication for the included studies were within the last five years, which might mitigate a potential effect of procedure-related inexperience that may be associated with earlier publications.

There are a few limitations to our study. First, there are several factors that were difficult to mitigate for when examining the data. The definition of ‘ICG positivity’ was not standardised between studies, and this is difficult to do with no established quantitative method and a significant degree of inter-user variability [82]. The intensity of fluorescence may be impacted by the dosage and concentration of ICG administered, the effects of which were not formally assessed in this study. Secondly, most of the data is derived from non-randomised studies, with a mixture of prospective and retrospective studies, and this is due to a dearth of high-quality randomised controlled studies. In general, it is acknowledged that it is difficult to conduct a robust randomised control trial with blinding of the surgeon due to the nature of ICG fluorescence, but a ubiquitous source of bias relates to the lack of blinding at the histopathological correlation level—that is, in all of the included studies in this meta-analysis, stratification of nodes as fluorescent/non-fluorescent took place prior to histopathological evaluation, and that ground truth correlation occurred with prior knowledge of the fluorescence result. Therefore, further high-quality studies are warranted to strengthen the robustness of our conclusions.

In conclusion, ICG-guided lymphadenectomy has the potential to be beneficial with lymph node harvest during oesophageal and gastric cancer resection, but the oncological benefits may be limited, and surgeons risk omitting a significant proportion of metastatic lymph nodes if this technique is solely relied upon. Further research should focus on circumventing some of the shortcomings associated with ICG, such as developing novel tracers that are more selective for metastases, or utilising other optical methods that do not rely on an exogenous tracer altogether.

## Supplementary Information

Below is the link to the electronic supplementary material.

Supplementary file1 (DOCX 20 KB)
