# Supplementary material for: The use of indocyanine green and near-infrared fluorescence in the detection of metastatic lymph nodes during oesophageal and gastric cancer resection: a systematic review and meta-analysis
Source: Surg Endosc. 2025 Apr 18;39(6):3525–38. doi: 10.1007/s00464-025-11703-7 (PMC12116833; doi:10.1007/s00464-025-11703-7)
Supplement: Supplementary file 1 — Supplementary file1 (DOCX 20 KB) [file 464_2025_11703_MOESM1_ESM.docx]

SUPPLEMENTARY TABLE 1

PRACTICAL ASPECTS OF ICG-GUIDED LYMPHADENECTOMY IN INCLUDED STUDIES

| **Study** | **ICG administration** | | | | **Fluorescence imaging system** | | **Method of lymph node examination** | | **Pathological examination** |
| --- | --- | --- | --- | --- | --- | --- | --- | --- | --- |
|  | *Total dose* | *Concentration* | *Method* | *Timing* | *Type* | *Manufacturer* | *Location of exam* | *Method of assessment* |  |
| Baiocchi, 2020 | 1-3mL | 2.5mg/mL | Submucosal or subserosal | 24h preop | Endoscopic | Karl Storz IMAGE1 Spies Camera; Stryker 1588 AIM platform with EMV modality; Novadaq PINPOINT camera system | Intracorporeal and ex-vivo (back table) | Verified visually by single surgeon | H&E |
|  |  | 0.5mg/mL |  | Intraop |  |  |  |  |  |
| Park, 2022 | 10mL | 0.01mg/mL | Subserosal | Intraop | Open field | Stryker SPY Elite NIR camera | Ex-vivo  (back table) only | Verified visually by single surgeon (implied but not explicitly stated) | H&E |
| Tuan, 2024 | 2.4mL | 1.25mg/mL | Submucosal | 24h preop | Endoscopic | Karl Storz 4K-S-Rubina Fluorescence system | Intracorporeal and ex-vivo (back table) | Verified visually by a single surgeon | H&E |
| Iwata, 2022 | 2mL | 0.5mg/mL | Submucosal | 24h preop | Endoscopic | Novadaq PINPOINT camera system | Intracorporeal and ex-vivo (back table) | Verified visually by a single surgeon (implied but not explicitly stated) | H&E |
| Jung, 2021 | 2.4mL | 0.625-1.25mg/mL | Submucosal | 24h preop | Endoscopic/  Robotic | Novadaq PINPOINT camera system (laparoscopic); Intuitive da Vinci Si or Xi Surgical System (robotic) | Intracorporeal and ex-vivo (back table) | Verified visually by a single surgeon (implied but not explicitly stated) | H&E |
| Roh, 2020 | 2.4mL | 0.625-1.25mg/mL | Submucosal | 24h preop | Endoscopic/ Robotic | Novadaq PINPOINT camera system (laparoscopic); Intuitive da Vinci Si or Xi Surgical System (robotic) | Intracorporeal and ex-vivo (back table) | Verified visually by a single surgeon (implied but not explicitly stated) | H&E |
| Chen, 2021 | 2mL | 1.25mg/mL | Submucosal | 24h preop | Endoscopic | Novadaq PINPOINT camera system | Intracorporeal and ex-vivo (back table) | Verified visually by surgical team, with secondary confirmation of recorded videos | H&E |
|  | 9mL | 0.5mg/mL | Subserosal | Intraop |  |  |  |  |  |
| Tian, 2025 | 2mL | 25mg/mL | Submucosal | 24h preop | Endoscopic/ Robotic | Novadaq PINPOINT camera system (laparoscopic); Intuitive da Vinci Xi Surgical System (robotic) | Intracorporeal and ex-vivo (back table) | Visual confirmation by one of two of the same surgeons | H&E and IHC |
| Wang, 2022 | 4mL | 1.25mg/mL | Submucosal | Intraop | Endoscopic | DPM-III-01, Zhuhai Dipu Medical Technology | Intracorporeal and ex-vivo (back table) | Verified visually by a single surgeon (implied but not explicitly stated) | H&E and IHC |
| Kim, 2024 | 2.4mL | 0.625-1.25mg/mL | Submucosal | 24h preop | Endoscopic/ Robotic | Novadaq PINPOINT camera system (laparoscopic); Intuitive da Vinci Xi Surgical System (robotic) | Intracorporeal and ex-vivo (back table) | Verified visually by a single surgeon (implied but not explicitly stated) | H&E |
| Chen, 2020 | 2mL | 0.625mg/mL | Submucosal | 24h preop | Endoscopic | Novadaq fluorescence surgical system | Intracorporeal and ex-vivo (back table) | Verified visually by surgical team, with secondary confirmation of recorded videos | H&E |
| Shiomi, 2023 | 2mL | 0.5mg/mL | Submucosal | 24h preop | Endoscopic | VISERA ELITE II Olympus Co | Intracorporeal and ex-vivo (back table) | Verified visually with consensus of 3+ surgeons | H&E |
| Huang, 2024 | 9mL | 0.5mg/mL | Subserosal | Intraop | Endoscopic | Karl Storz IMAGE1 CONNECT fluorescence system | Intracorporeal and ex-vivo (back table) | Verified visually by surgical team, with secondary confirmation of recorded videos | H&E |
| Cianchi, 2020 | 2mL | 1.25mg/mL | Submucosal | 24h preop | Robotic | Intuitive da Vinci Si Surgical System | Intracorporeal and ex-vivo (back table) | Verified visually by a single surgeon | H&E |
| Lee, 2022 | 2.4mL | 0.625-1.25mg/mL | Submucosal | 24h preop | Endoscopic/ Robotic | Novadaq PINPOINT camera system (laparoscopic); Intuitive da Vinci Si or Xi Surgical System (robotic) | Intracorporeal and ex-vivo (back table) | Verified visually by a single surgeon | H&E |

*H&E: Haemoxylin & Eosin; IHC: Immunohistochemistry*
